# Supplementary material for: Crystal structures of herbicide-detoxifying esterase reveal a lid loop affecting substrate binding and activity
Source: Nat Commun. 2023 Jul 19;14:4343. doi: 10.1038/s41467-023-40103-5 (PMC10356948; doi:10.1038/s41467-023-40103-5)
Supplement: Supplementary file 1 — Supplementary Information [file 41467_2023_40103_MOESM1_ESM.pdf]

Crystal structures of herbicide-detoxifying esterase reveal a lid loop affecting substrate binding and activity

Liu *et al*.

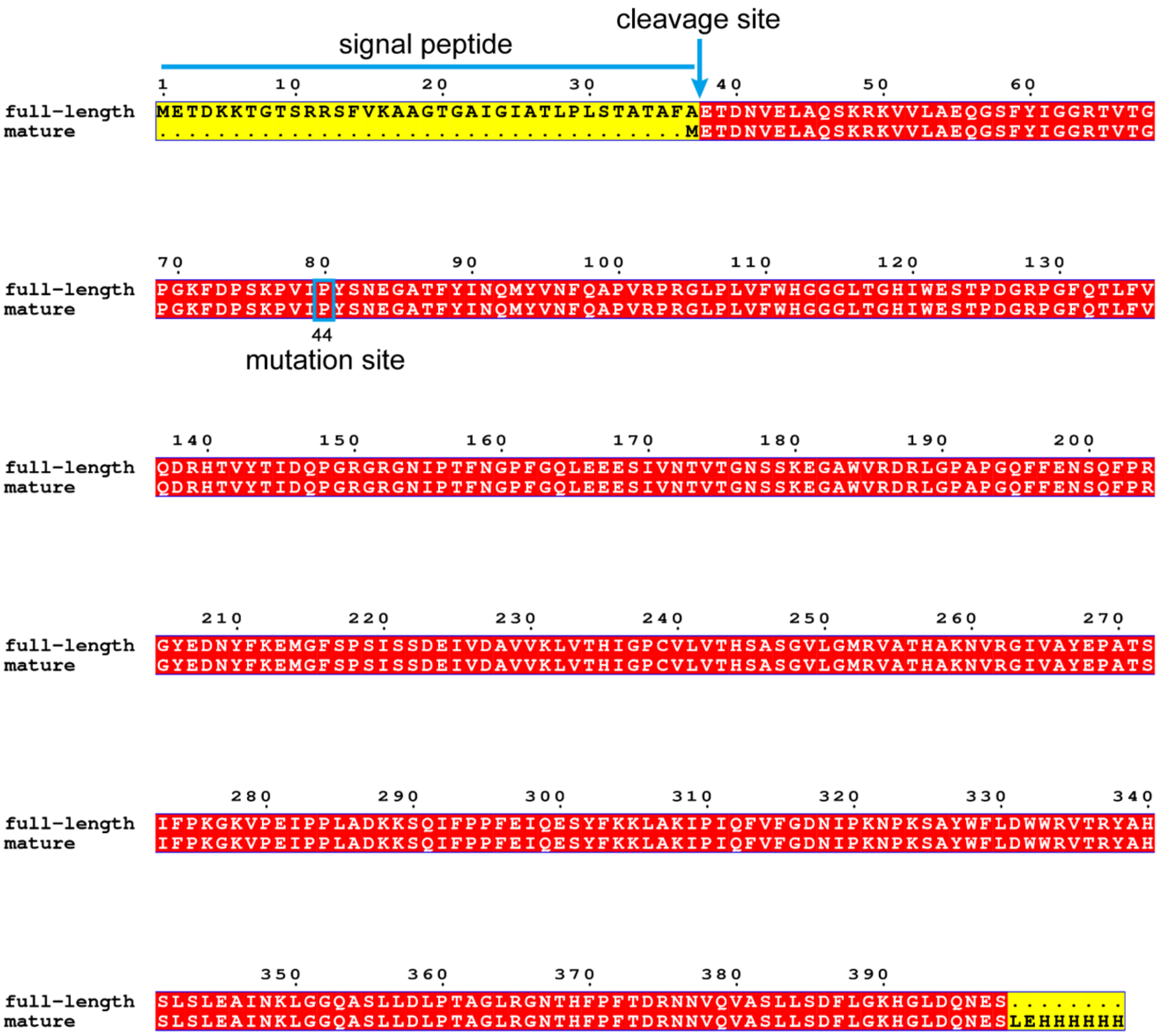

Supplementary Figure 1. Sequence alignment of full-length SulE and mature SulE. The signal peptide is marked with a cyan line, the arrow points to the signal peptide cleavage site, which is located between Ala37 and Glu38. Marked with a cyan box is the mutation position P44R or P80R.

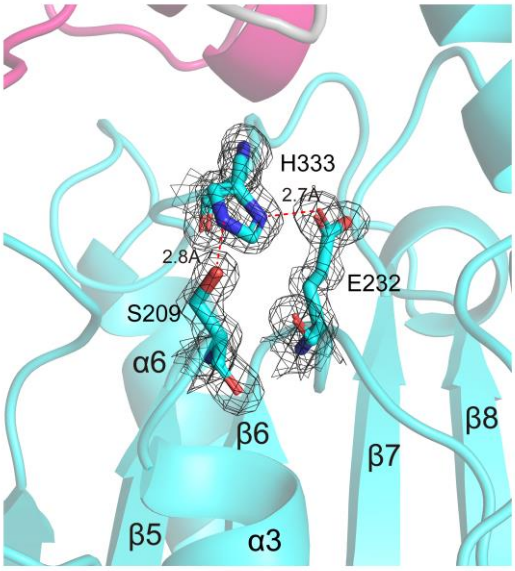

Supplementary Figure 2. Residues corresponding to catalytic triads in SulE. The electron density maps (2Fo_Fc) for the triad are light black and contoured at 1.0 σ. The red dotted lines indicate the hydrogen bonds. Secondary structure elements are labeled.

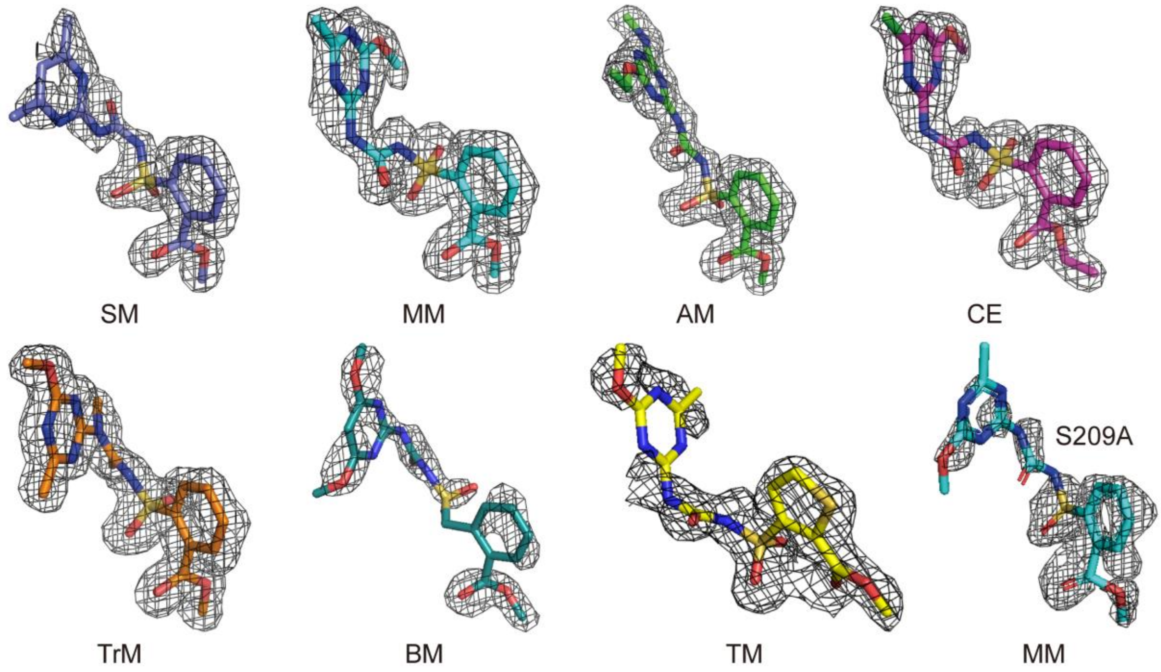

Supplementary Figure 3. Electron density maps of seven sulfonylureas. The 1st-7th (from left to right, top to bottom) are the electron density maps of the substrates observed at the S209A/H333A active site, and the last one is the electron density map of the MM observed at the S209A active site. The corresponding electron density maps (2Fo_Fc) are light black and contoured at 1.0 σ.

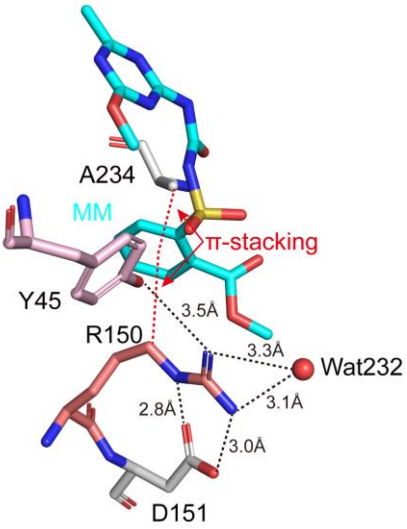

Supplementary Figure 4. A detailed interaction network of Arg150 in MM-bound S209A/H333A. Substrate molecule MM, Arg150, Tyr45 and the other two residues (Ala234 and Asp151) are highlighted in different colors. Water molecule Wat232 is shown in red sphere. Hydrogen bonds are indicated by black dashed lines. The red dotted line indicates the π-stacking interaction.

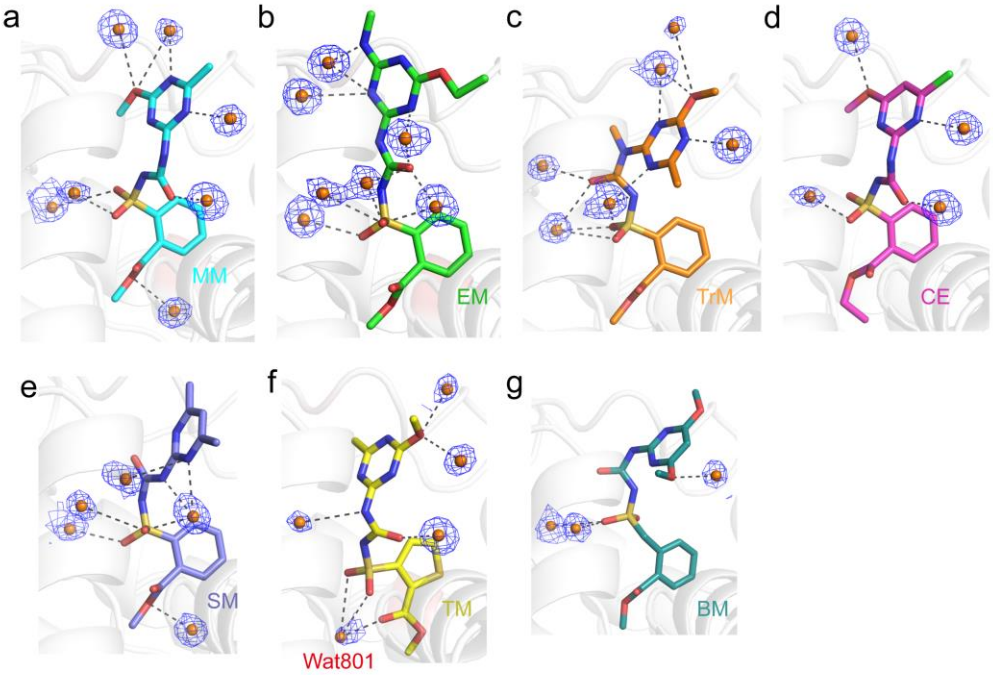

Supplementary Figure 5. Water molecule that form hydrogen bonds with the substrate at the active site. a-g the active sites correspond to a-g in Fig. 3. The water molecules are displayed as orange spheres. The 2Fo_Fc electron density maps of water molecules were contoured at 1.0 σ in blue color. The black dashed lines indicate hydrogen bonds. In the S209A/H333A-TM complex structure (f), water molecule Wat801 that form hydrogen bond with ester O atom is labled in red.

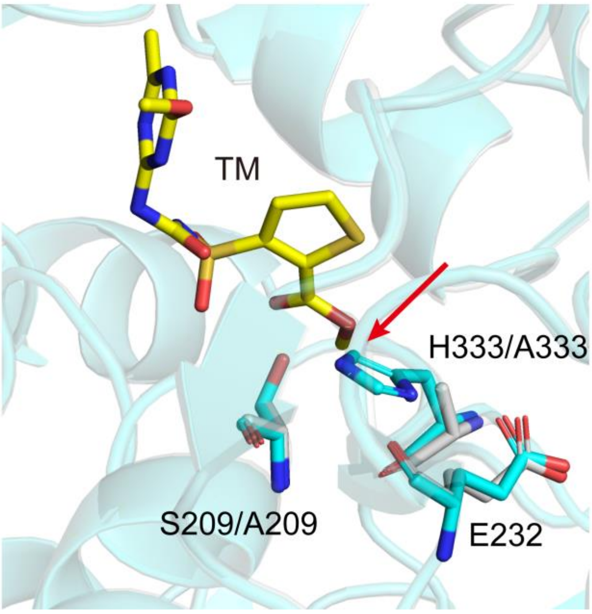

Supplementary Figure 6. Analysis of the conformation of TM at the S209A/H333A active site. Apo-SulE and S209A/H333A are shown in cyan and white transparent cartoons, respectively, and the catalytic triads are shown as sticks. Superimposition of the structures of apo-SulE and S209A/H333A-TM complex showed that the oxymethyl group of TM and the imidazole ring of H333 side chain are incompatible with each other, indicating that TM is not bound in the correct catalytic form at the active site of S209A/H333A.

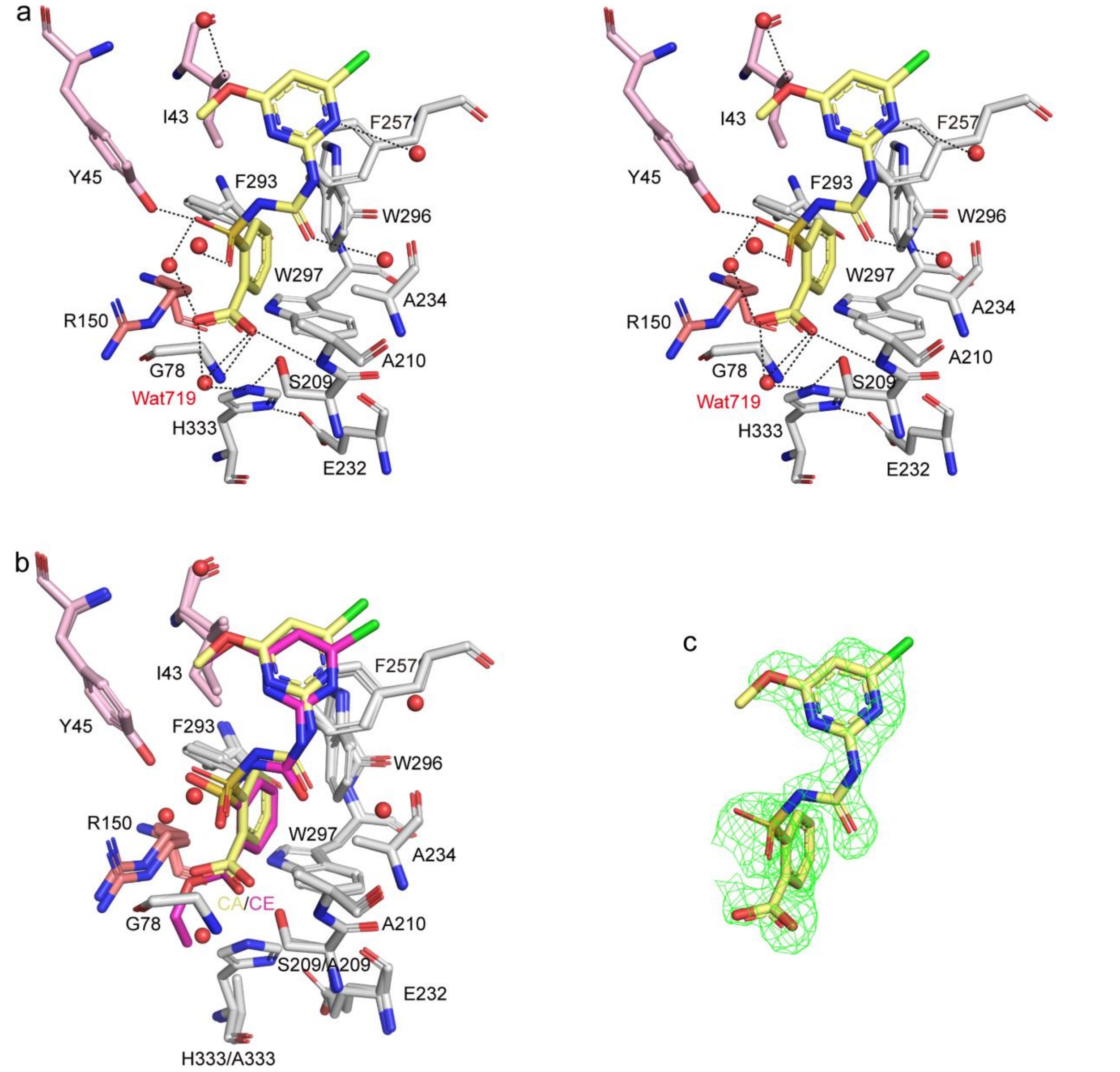

Supplementary Figure 7. Crystal structure of WT SulE in complex with product CA. a Stereo view of detailed interaction network between SulE and CA. **b** Superposition of active sites of SulE-CA and S209A/H333A-CE complex structures. **c** The electron density of CA. The water molecules are shown as red spheres, and the 2Fo_Fc electron density map of CA was contoured at 1.0 σ in green color. Hydrogen bonds are shown in black dashed lines. The probable deacylating water molecule is labled in red.

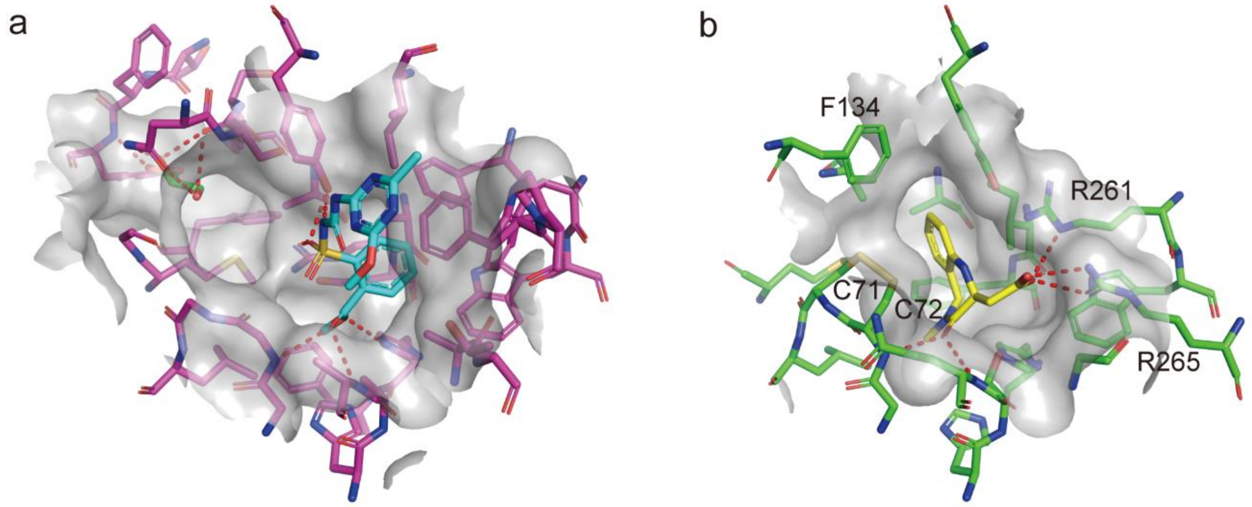

Supplementary Figure 8. The substrate-binding pockets of SulE (a) and esterase 713 (b). MM and IBA are depicted in cyan and orange, respectively. Hydrogen bonds are shown as red dashes.

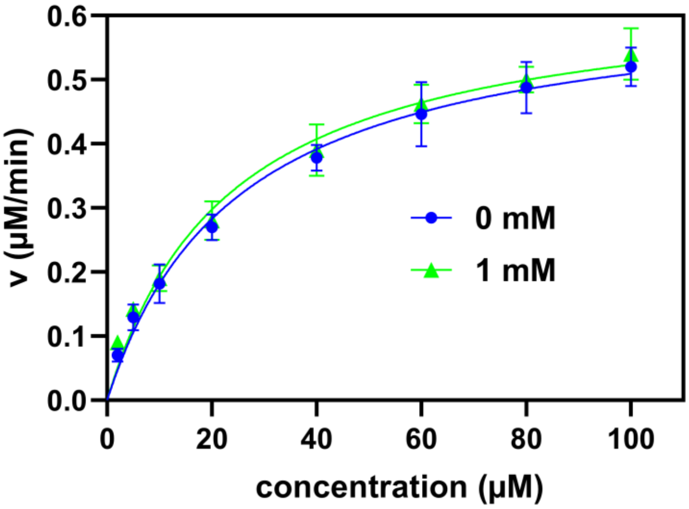

Supplementary Figure 9. Inhibition effect of product CA on SulE. Michaelis-Menten kinetic experiment was performed, testing product CA at an inhibitor concentration of 1mM, while varying CE concentrations from 2 to 100 μM. Mean values for n=3 replicates ± SD are shown.

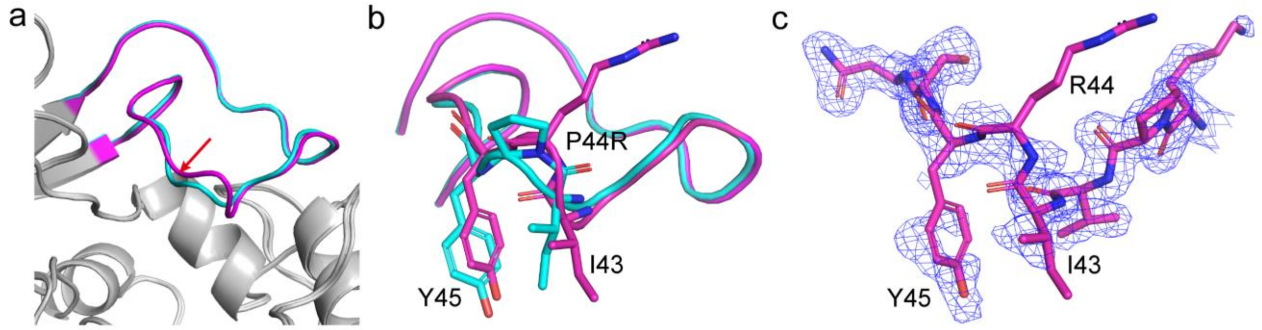

Supplementary Figure 10. Structural comparison analysis between WT apo-SulE and apo-P44R. **a** Superposition structures of apo-P44R and WT apo-SulE. The well superimposed part is colored gray. The varied lid loop is colored cyan for SulE and magenta for P44R. **b** Detailed comparative analysis of the positions of the three residues I43, P44 (R44), and Y45 in the lid loop. **c** The electron density maps of residues 40-47 located in the lid loop of P44R. The 2Fo_Fc electron density map contoured at 1.0 σ level is shown as blue mesh.

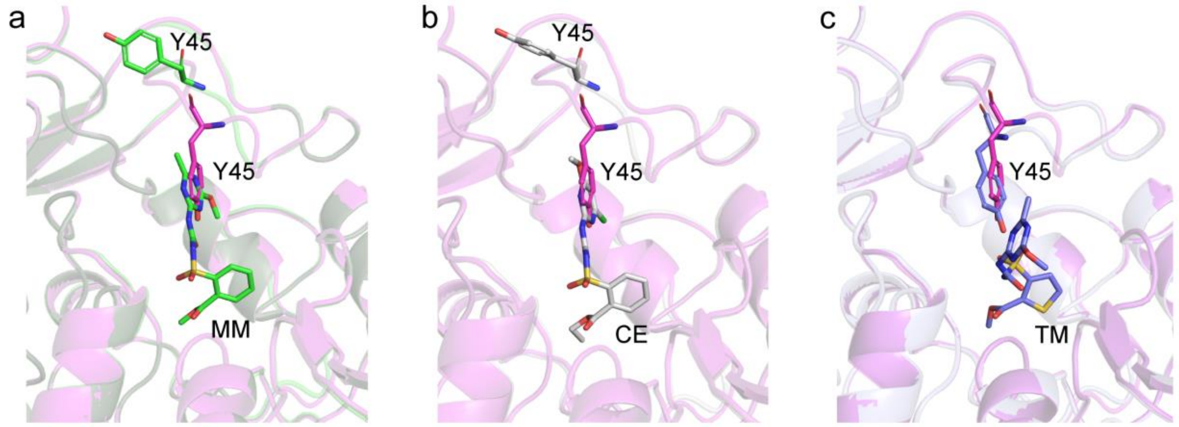

Supplementary Figure 11. Superposition structures of apo-P44R and P44R-bound substrate. a Cartoon representation of the active site of P44R in the absence (magenta) or in the presence of MM (green). **b** Cartoon representation of the active site of P44R in the absence (magenta) or in the presence of CE (white). **c** Cartoon representation of the active site of P44R in the absence (magenta) or in the presence of MM (light blue). The cartoon is adjusted to 80% transparent. Mutation of Pro44 to arginine makes the lid loop region more flexible. The binding of MM or CE push the lid loop away from the active site. However, the binding of TM does not induce a conformational change in the lid loop. The major change caused by MM or CE binding in the active site is observed in Tyr45, whose main chain Cα was shifted by about 3.0 Å and the side chain was rotated by 90 degrees.

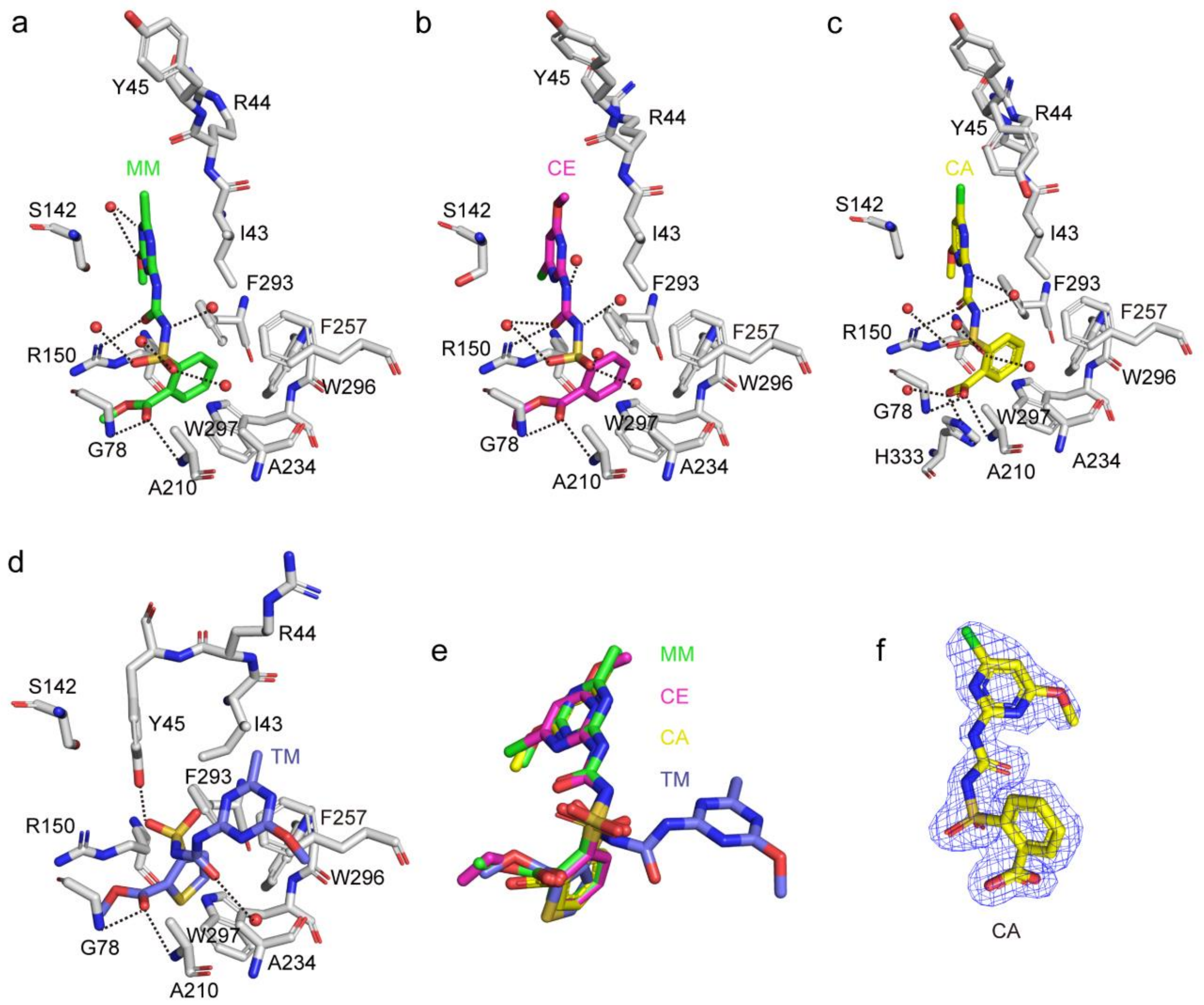

Supplementary Figure 12. Structural analysis of mutant P44R. a, b, d The substrate binding pocket of P44R/S209A/H333A with MM and TM, respectively. **c** The substrate binding pocket of P44R/S209A with product CA. **e** Superposition of MM, CE, TM and CA. **f** The electron density of CA observed at the active site of P44R/S209A. The water molecules are shown as red spheres, and the 2Fo_Fc electron density map of CA was contoured at 1.0 σ in blue color. Hydrogen bonds are shown in black dashed lines.

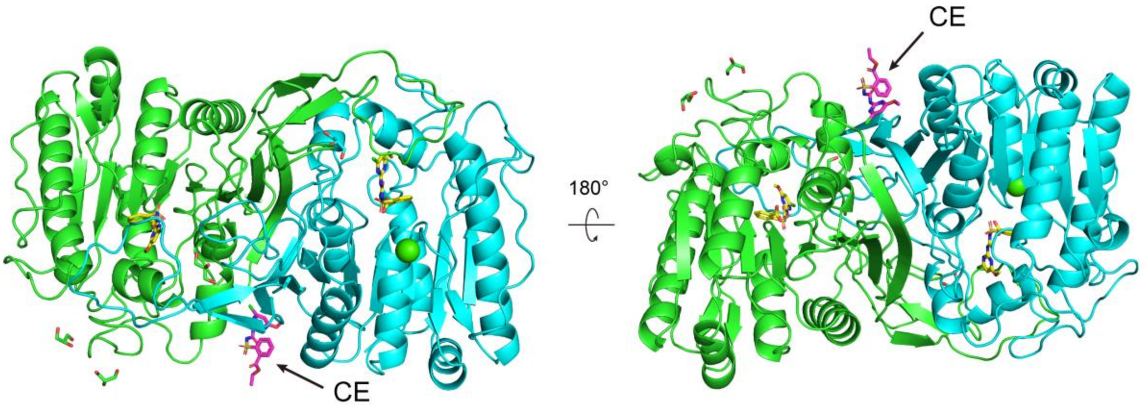

Supplementary Figure 13. P44R/S209A-CA complex structure. The CA bound in the active site is shown as yellow stick and the CE in the inactive site is shown as magentas.

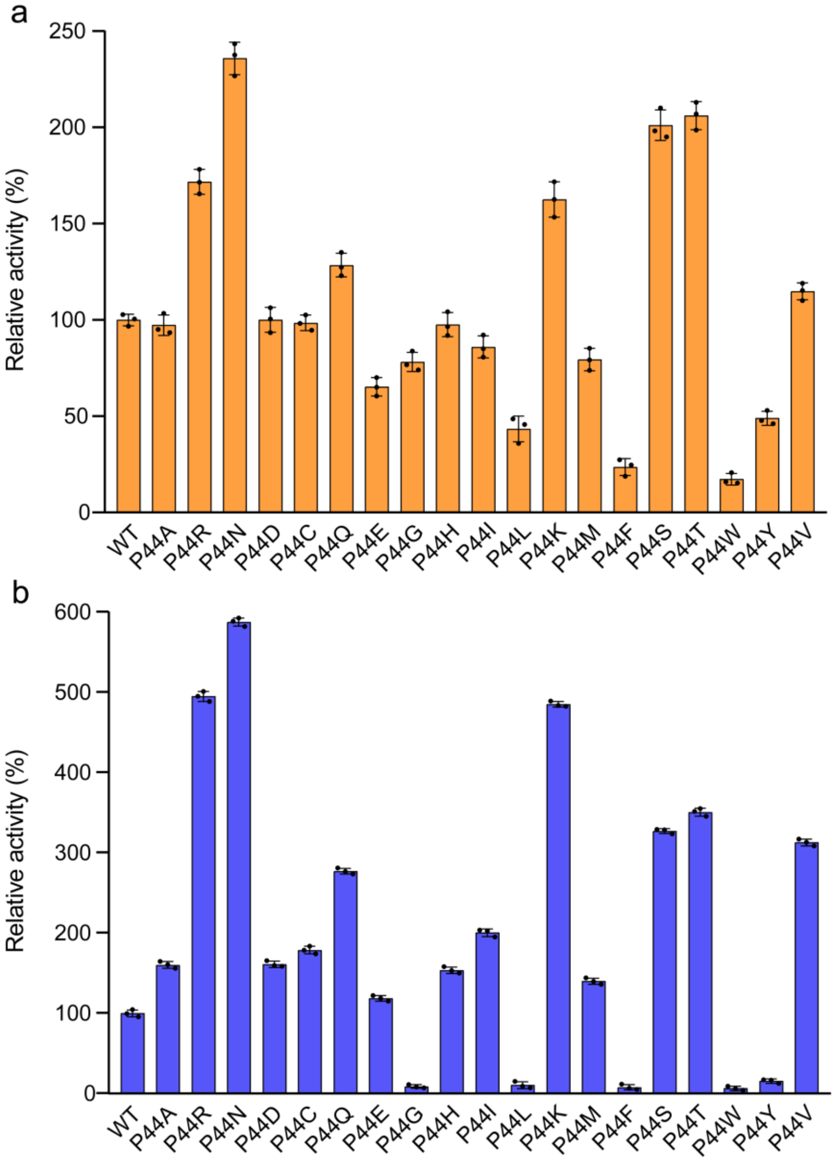

Supplementary Figure 14. Pro44 saturation mutation. a The relative enzyme activity of the variants to MM. **b** The relative enzyme activity of the variants to CE. Data are presented as mean values ± SD,

n = 3. Error bars represent the standard deviation from three repeats. Source data are provided as a Source Data file.

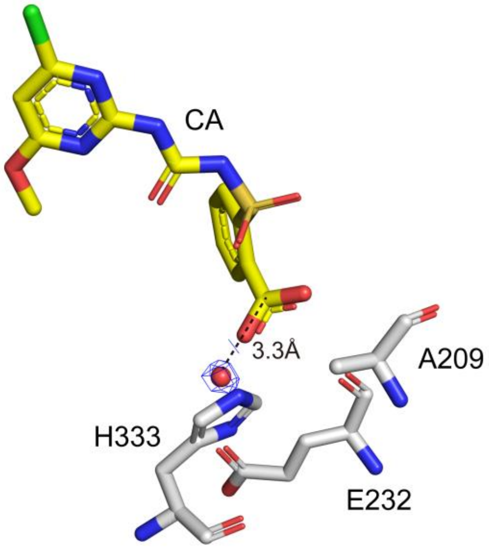

Supplementary Figure 15. The active site of P44R/S209A-CA complex structure. The catalytic triad is shown as white sticks and CA is presented as yellow stick. A water molecule in the active site is highlighted in red. The 2Fo_Fc electron density map of CA was contoured at 1.0 σ in blue color. The distance between the water molecule and the carbonyl carbon atom is indicated by the black dotted line.

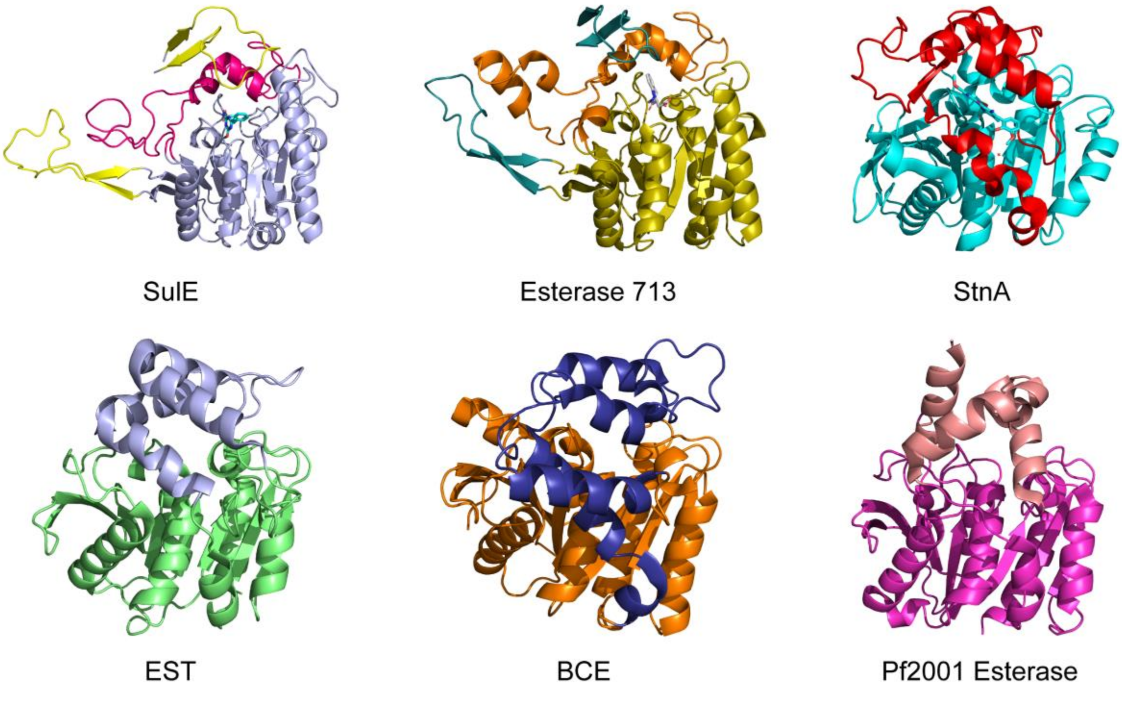

Supplementary Figure 16. Comparison of the structure of esterase SulE and its homologs. The cap domains of SulE (PDB: 7Y0L), esterase 713 (PDB: 1QLW), StnA (PDB: 5HDP), EST

(PDB:1ZOI), BCE (PDB: 5OLU) and Pf2001 Esterase (PDB: 5G59) are highlighted in hot pink, orange, red, light blue, deep blue and salmon, respectively. The lid loop of SulE and esterase 713 is highlighted in yellow and deep teal.

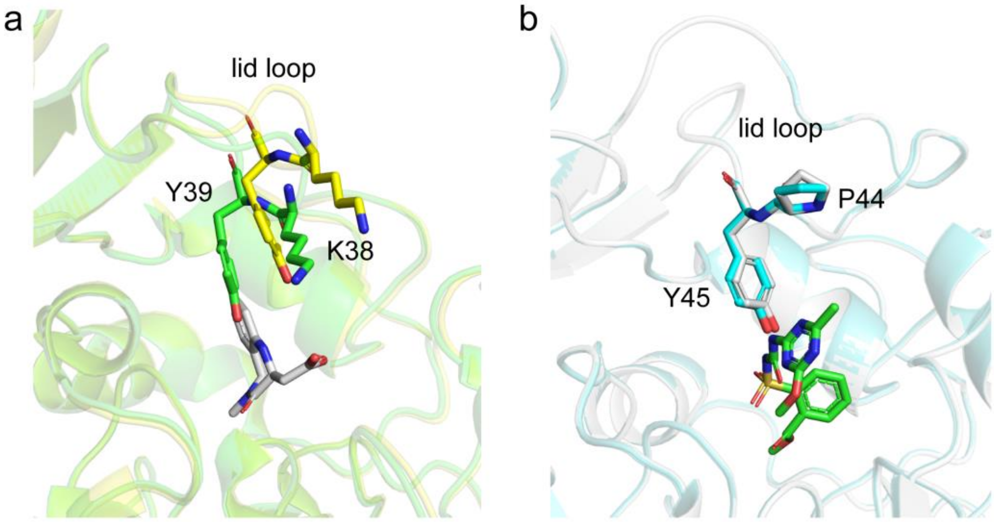

Supplementary Figure 17. Conformational changes of the lid loop of esterase 713 and SulE when the product or substrate is combined. a Superposition of structures of esterase 713 (green) and esterase 713-IBA complex (yellow). **b** Superposition of structures of SulE (gray) and S209A-MM complex (cyan). Residues selected in the lid loop are shown as sticks.

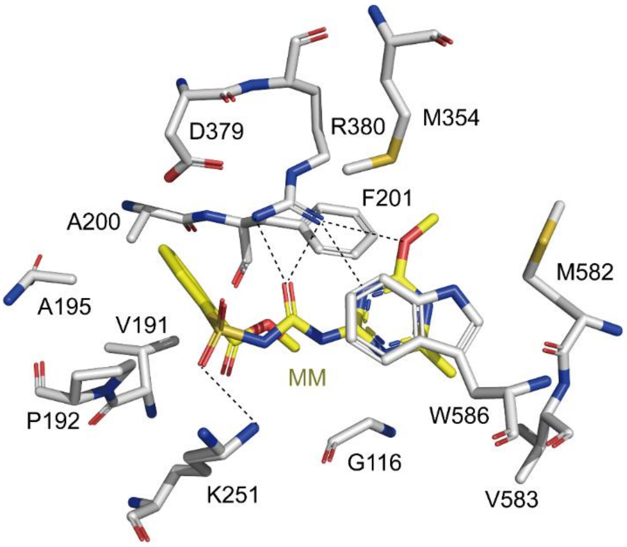

Supplementary Figure 18. MM binding sites in *Sc*AHAS. MM is colored with C atoms in yellow. Other residues are colored with C atoms in white. Hydrogen bonds are shown in black dashed lines.

Supplementary Table 1. Data collection and structure refinement statistics

	SulE	SulE-CA	S209A-MM	S209A/H333A-MM	S209A/H333A-CE
**PDB ID**	**8GP0**	**8GOL**	**7Y0L**	**8IVN**	**8IW3**
**Data Collection**					
Wavelength (Å)	0.9791	0.9792	0.9792	0.9792	0.9792
Space group	$P2_1$	$P2_1$	$P2_1$	$P2_1$	$P2_1$
Cell dimensions					
a, b, c (Å)	51.37, 139.19, 57.91	55.42, 166.66, 76.96	51.22, 139.86, 58.10	51.23, 139.73, 58.15	51.36, 140.03, 58.26
α, β, γ (°)	90.00, 101.29, 90.00	90.00, 109.74, 90.00	90.00, 101.60, 90.00	90.00, 101.42, 90.00	90.00, 101.41, 90.00
Resolution (Å)	50.00-1.46 (1.49-1.46)	49.78-1.60 (1.69-1.60)	36.06-1.29 (1.31-1.29)	42.03-1.50 (1.54-1.50)	36.1-1.56 (1.60-1.56)
Observed reflections	854841 (22025)	1153798 (146123)	1278954 (50484)	758414 (27575)	527071 (30425)
Unique reflections	131514 (4798)	168217 (22718)	193359 (9349)	126161 (8460)	113711 (8347)
R_{pim}	0.037 (0.204)	0.028 (0.135)	0.031 (0.241)	0.040 (0.395)	0.044 (0.423)
$CC_{1/2}$	0.998 (0.874)	0.998 (0.941)	0.993 (0.925)	0.992 (0.698)	0.996 (0.648)
Completeness (%)	95.26 (75.57)	98.00 (90.80)	96.7 (93.4)	98.9 (90.0)	99.5 (99.0)
$I/\delta(I)$ [a]	20.8 (1.9)	14.8 (4.6)	28.51 (2.73)	16.4 (1.9)	10.1 (1.7)
Multiplicity	6.5 (4.6)	6.9 (6.4)	6.6 (5.4)	6.0 (3.3)	4.6 (3.6)
Wilson B-factor	14.88	15.47	12.38	13.46	18.21
**Refinement**					
R_{work}	0.1520	0.1512	0.1542	0.1635	0.1701
R_{free}	0.1693	0.1811	0.1672	0.1797	0.1851
No. of non-H atoms					
Protein	5511	11033	5602	5488	5542
Ligands	56	124	82	110	32
Water	893	1456	929	851	811
Clashscore	2.90	1.77	4.36	2.54	1.27
B-factors (Å^2)					
Protein	17.89	17.37	16.27	16.20	20.79
Ligand	27.43	31.95	29.51	22.62	20.00
Water	30.83	27.70	29.09	28.54	32.97
R.m.s.deviations					
Bond lengths (Å)	0.008	0.006	0.007	0.009	0.023
Bond angles (°)	1.24	0.83	0.98	1.24	1.20
Ramachandran plot (%)					
Favoured	97.70	97.27	97.27	97.55	97.12
Allowed	2.30	2.73	2.73	2.45	2.88
outliers	0	0	0.00	0.00	0.00

Continued

	S209A/H333A-SM	S209A/H333A-EM	S209A/H333A-TrM	S209A/H333A-TM	S209A/H333A-BM
**PDB ID**	**8IW6**	**8IVS**	**8IVT**	**8J7J**	**8J7G**
**Data Collection**					
Wavelength (Å)	0.9791	0.9792	0.9792	0.9787	0.9787
Space group	$P2_1$	$P2_1$	$P2_1$	$P2_1$	$P2_1$
Cell dimensions					
a, b, c (Å)	50.97, 139.93, 58.03	51.04, 140.06, 58.21	51.14, 139.60, 58.19	51.15, 139.55, 57.95	51.05, 139.17, 57.92
α, β, γ (°)	90.00, 101.57, 90.00	90.00, 101.49, 90.00	90.00, 101.43, 90.00	90.00, 101.68, 90.00	90.00, 108.25, 90.00
Resolution (Å)	24.97-1.44 (1.48-1.44)	36.01-1.52 (1.56-1.52)	50.13-1.42 (1.49-1.42)	19.94-1.54 (1.57-1.54)	19.99-1.63 (1.66-1.63)
Observed reflections	783864 (9543)	570157 (22602)	631092 (43761)	522261 (7770)	457653 (12396)
Unique reflections	132554 (5887)	121276 (7837)	139811 (13669)	113305 (3502)	92883 (4472)
R_{pim}	0.024 (0.429)	0.030 (0.319)	0.030 (0.245)	0.024 (0.393)	0.035 (0.274)
$CC_{1/2}$	0.999 (0.635)	0.998 (0.774)	0.998 (0.852)	0.999 (0.791)	0.998 (0.776)
Completeness (%)	92.6 (55.9)	98.9 (86.6)	92.8 (62.4)	96.8 (60.1)	97.5 (94.4)
$I/\delta(I)$	18.1 (1.3)	14.1 (2.0)	14.4 (2.5)	16.9 (1.9)	12.3 (2.2)
Multiplicity	5.9 (1.6)	4.7 (2.9)	4.5 (3.2)	4.6 (2.2)	4.9 (2.8)
Wilson B-factor	13.44	15.86	13.48	15.24	16.26
**Refinement**					
R_{work}	0.1666	0.1567	0.1614	0.1495	0.1625
R_{free}	0.1800	0.1713	0.1737	0.1679	0.1890
No. of non-H atoms					
Protein	5477	5477	5488	5472	5488
Ligands	82	88	86	82	88
Water	861	843	891	800	663
Clashscore	1.64	2.82	3.55	1.92	2.18
B-factors (Å^2)					
Protein	16.36	18.20	16.66	18.17	19.28
Ligand	25.90	20.55	19.62	32.38	39.79
Water	28.12	30.31	29.16	29.70	30.26
R.m.s.deviations					
Bond lengths (Å)	0.010	0.009	0.007	0.006	0.007
Bond angles (°)	1.36	1.28	1.22	0.85	0.86
Ramachandran plot (%)					
Favoured	97.41	97.55	97.26	97.12	97.69
Allowed	2.59	2.45	2.74	2.88	2.31
outliers	0.00	0.00	0.00	0.00	0.00

Continued

	P44R	P44R/S209A-CA	P44R/S209A/H333A-MM	P44R/S209A/H333A-CE	P44R/S209A/H333A-TM
**PDB ID**	**8GOY**	**7YD2**	**8IVM**	**8IVE**	**8J7K**
**Data Collection**					
Wavelength (Å)	0.9792	0.9792	0.9792	0.9792	0.9787
Space group	$P2_1$	$P2_1$	$P2_1$	$P2_1$	$P2_1$
Cell dimensions					
a, b, c (Å)	51.353, 139.450, 58.125	51.31, 140.26, 58.19	51.16, 139.67, 58.16	51.08, 140.08, 58.22	51.09, 139.69, 57.90
α, β, γ (°)	90.00, 101.67, 90.00	90.00, 101.71, 90.00	90.00, 101.56, 90.00	90.00, 101.67, 90.00	90.00, 108.67, 90.00
Resolution (Å)	47.31-1.78 (1.85-1.78)	70.13-1.61 (1.65-1.61)	47.18-1.32 (1.35-1.32)	57.01-1.44 (1.51-1.44)	19.96-1.36 (1.38-1.36)
Observed reflections	471995 (8520)	640236 (31187)	851550 (28627)	834883 (88531)	724162 (11067)
Unique reflections	74840 (3200)	102138 (7066)	182251 (11288)	145171 (21201)	154352 (4302)
R_{pim}	0.048 (0.509)	0.031 (0.192)	0.025 (0.363)	0.035 (0.311)	0.018 (0.191)
$CC_{1/2}$	0.997 (0.572)	0.997 (0.898)	0.999 (0.757)	0.998 (0.778)	0.999 (0.908)
Completeness (%)	98.30 (73.4)	98.8 (92.6)	97.6 (81.8)	99.8 (100.0)	94.1 (52.7)
$I/\delta(I)$	11.7 (1.2)	14.6 (3.2)	15.3 (1.8)	13.0 (2.5)	20.7 (2.8)
Multiplicity	6.3 (2.7)	6.3 (4.4)	4.7 (2.5)	5.8 (4.2)	4.7 (2.6)
Wilson B-factor	17.72	20.56	12.94	14.77	13.09
**Refinement**					
R_{work}	0.1585	0.1646	0.1601	0.1771	0.1512
R_{free}	0.1853	0.1812	0.1709	0.1961	0.1659
No. of non-H atoms					
Protein	5523	5517	5501	5550	5496
Ligands	50	86	110	12	82
Water	592	674	788	786	789
Clashscore	2.36	1.82	3.26	2.37	1.64
B-factors ($Å^2$)					
Protein	18.25	23.16	16.37	18.03	15.97
Ligand	29.78	27.99	18.79	20.00	34.57
Water	29.72	35.16	28.13	29.36	28.21
R.m.s.deviations					
Bond lengths (Å)	0.008	0.018	0.009	0.020	0.008
Bond angles (°)	1.19	1.28	1.27	1.25	0.94
Ramachandran plot (%)					
Favoured	96.83	97.26	97.26	97.55	97.84
Allowed	2.74	2.74	2.74	2.45	2.16
outliers	0.43	0.00	0.00	0.00	0.00

Values for the outmost resolution shell are given in parentheses

Supplementary Table 2. Structure homolog hits of SulE were searched by Dali sever

Protein name	ESTHER family	PDB-chain	Z score	RMSD (Å)	LALI	NRES	% ID
uncharacterized protein from *Parabacteroides distasonis*	Bacterial_esterase	4Q34-A	41.0	2.0	310	321	37
Esterase 713 from *Alcaligenes*	Bacterial_esterase	1QLW-A	37.4	2.0	297	318	34
Streptonigrin methylesterase A (StnA) from *Streptomyces*	6_AlphaBeta_hydrolase	5HDF-B	19.1	3.0	217	320	12
Uncharacterized protein from *Thermoplasma acidophilum*	CIB-CCG1-interacting-factor-B	3BDI-A	18.8	3.0	200	207	16
Stereoselective Esterase from *Pseudomonas putida* IFO12996	Haloperoxidase	1ZOI-A	18.6	2.9	202	275	21
EstN2 from Candidatus *Nitrososphaera gargensis*	6_AlphaBeta_hydrolase	5A62-A	18.3	2.6	201	272	19
Carboxyl esterase (BCE) from *Bacillus coagulans*	Monoglyceridelipase_lysophospholip	5O7G-A	18.0	2.4	201	310	16
Pf2001 esterase from *Pyrococcus furiosus*	AlphaBeta_hydrolase	5G59-A	17.9	2.8	202	274	12
putative aryl esterase from *Burkholderia cenocepacia*	6_AlphaBeta_hydrolase	4X00-A	17.9	2.6	195	273	17
putative hydrolase from *Bacillus subtilis*	6_AlphaBeta_hydrolase	2R11-D	17.8	3.0	196	288	16
human soluble epoxide hydrolase	Epoxide_hydrolase	4HAI-A	17.8	3.3	214	548	14

Supplementary Table 3. Primers used in this study

Primer	DNA Sequence (5´ to 3´)
sulE-F	TAAGAAGGAGATATACATATGGAAACTGACAACGTGGAGCTTGC
sulE-R	GTGGTGGTGGTGGTGCTCGAGGCTTTCGTTCTGATCTAAGCCG
Y45A-F	AAGCCGGTAATTCCAGCTTCCAACGAAGGTGC/
Y45A-R	GCACCTTCGTTGGAAGCTGGAATTACCGGCTT
I43A-F	AAAGCCGGTAGCTCCATATTCCAACGAAGGTG
I43A-R	GGAATATGGAGCTACCGGCTTTGACGGATCGA
G78A-F	TTGGCATGGGGCCGGACTAACCGGCCATATCT
G78A-R	GGTTAGTCCGGCCCCATGCCAAAAGACTAGA
R150A-F	TAGTCGATCTGCAACCCACGCTCCTTCTTTAC
R150A-R	AGCGTGGGTTGCAGATCGACTAGGGCCCGCTC
S209A-F	CCGGAAGCCGCATGGGTCACCAGAACACAAG
S209A-R	GGTGACCCATGCGGCTTCCGGAGTACTGGGC
A210Q-F	GACCCATTCGCAGTCCGGAGTACTGGGCATGCG
A210Q-R	GTACTCCGGACTGCGAATGGGTCACCAGAACAC
E232A-F	TGTCGCAGGCGCATAAGCAACGATCCCCCTC
E232A-R	GTTGCTTATGCGCCTGCGACAAGTATCTTTC
A234S-F	TTATGAGCCTTCGACAAGTATCTTTCCCAAA
A234S-R	ATACTTGTCGAAGGCTCATAAGCAACGATCC
F257A-F	GTCGCAAATTGCCCCGCCGTTCGAGATCCAGG
F257A-R	GAACGGCGGGGCAATTTGCGACTTTTTATCGG
F257W-F	GTCGCAAATTTGGCCGCCGTTCGAGATCCAGG
F257W-R	CGAACGGCGGCCAAATTTGCGACTTTTTATCG
F293A-F	GCCTATTGGTCGTTGGACTGGTGGAGAGTCAC
F293A-R	CCAGTCCAAGGCCCAATAGGCGGATTTAGGGT
W296A-F	GTTCTTGGACGCGTGGAGAGTCACTCGCTACG
W296A-R	GACTCTCCACGCGTCCAAGAACCAATAGGCGG
W297A-F	CTTGGACTGGGCGAGAGTCACTCGCTACGCTC
W297A-R	AGTGACTCTCGCCCAGTCCAAGAACCAATAGG
H333A-F	GAATGGAAAAGCCGTGTTGCCGCGAAGTCCCG
H333A-R	CGGCAACACGGCTTTTCCATTCACCGACCGGA

Continued

Primer	DNA Sequence (5′ to 3′)
P33G-F	AGAACAGTAACCGGGGGTGGAAAATTCGATCC
P33G-R	CCCGGTTACTGTTCTGCCCCCGATG
P38G-F	CCTGGAAAATTCGATGGGTCAAAGCCGGTAAT
P38G-R	ATCGAATTTTCCAGGCCCGGTTACTGTT
P41G-F	TTCGATCCGTCAAAGGGGGTAATTCCATATTC
P41G-R	CTTTGACGGATCGAATTTTCCAGGC
Y45F-F	AAGCCGGTAATTCCATTTTCCAACGAAGGTGC
Y45F-R	TGGAATTACCGGCTTTGACGGATCG
G32A-F	GGCAGAACAGTAACCGCGCCTGGAAAATTCGA
G32A-R	GGTTACTGTTCTGCCCCCGATGTAG
G34A-F	ACAGTAACCGGGCCTGCAAAATTCGATCCGTC
G34A-R	AGGCCCGGTTACTGTTCTGCCCC
G50A-F	CATATTCCAACGAAGCTGCCACGTTTTATAT
G50A-R	TTCGTTGGAATATGGAATTACCGGC
P44H-F	CAAAGCCGGTAATTCACTATTCCAACGAAGGT
P44Q-F	CAAAGCCGGTAATTCAGTATTCCAACGAAGGT
P44L-F	CAAAGCCGGTAATTCTATATTCCAACGAAGG
P44Y-F	TCAAAGCCGGTAATTTACTATTCCAACGAAGGT
P44C-F	TCAAAGCCGGTAATTTGCTATTCCAACGAAGGT
P44D-F	TCAAAGCCGGTAATTGACTATTCCAACGAAGGT
P44F-F	TCAAAGCCGGTAATTTTCTATTCCAACGAAGGT
P44M-F	TCAAAGCCGGTAATTATGTATTCCAACGAAGGT
P44W-F	TCAAAGCCGGTAATTTGGTATTCCAACGAAGGT
P44E-F	TCAAAGCCGGTAATTGAATATTCCAACGAAGG
P44V-F	TCAAAGCCGGTAATTGTATATTCCAACGAAGG
P44T-F	TCAAAGCCGGTAATTACATATTCCAACGAAG
P44S-F	TCAAAGCCGGTAATTTCATATTCCAACGAAG
P44I-F	TCAAAGCCGGTAATTATATATTCCAACGAAGG
P44I-R	AATTACCGGCTTTGACGGATCG
P44G-F	TCAAAGCCGGTAATTGGATATTCCAACGAAGG
P44A-F	TCAAAGCCGGTAATTGCATATTCCAACGAAGG
P44N-F	TCAAAGCCGGTAATTAACTATTCCAACGAAGGT
