# Supplementary material for: Crystal structures of herbicide-detoxifying esterase reveal a lid loop affecting substrate binding and activity
Source: Nat Commun. 2023 Jul 19;14:4343. doi: 10.1038/s41467-023-40103-5 (PMC10356948; doi:10.1038/s41467-023-40103-5)
Supplement: Supplementary file 3 — Reporting Summary [file 41467_2023_40103_MOESM3_ESM.pdf]

nature portfolio

Corresponding author(s): Tingting Ran
Jian He

Last updated by author(s): Jun 27, 2023

Reporting Summary

Nature Portfolio wishes to improve the reproducibility of the work that we publish. This form provides structure for consistency and transparency in reporting. For further information on Nature Portfolio policies, see our Editorial Policies and the Editorial Policy Checklist.

Statistics

For all statistical analyses, confirm that the following items are present in the figure legend, table legend, main text, or Methods section.

n/a	Confirmed	
☐	☒	The exact sample size (*n*) for each experimental group/condition, given as a discrete number and unit of measurement
☐	☒	A statement on whether measurements were taken from distinct samples or whether the same sample was measured repeatedly
☐	☒	The statistical test(s) used AND whether they are one- or two-sided<br>*Only common tests should be described solely by name; describe more complex techniques in the Methods section.*
☒	☐	A description of all covariates tested
☒	☐	A description of any assumptions or corrections, such as tests of normality and adjustment for multiple comparisons
☐	☒	A full description of the statistical parameters including central tendency (e.g. means) or other basic estimates (e.g. regression coefficient) AND variation (e.g. standard deviation) or associated estimates of uncertainty (e.g. confidence intervals)
☐	☒	For null hypothesis testing, the test statistic (e.g. *F*, *t*, *r*) with confidence intervals, effect sizes, degrees of freedom and *P* value noted<br>*Give P values as exact values whenever suitable.*
☒	☐	For Bayesian analysis, information on the choice of priors and Markov chain Monte Carlo settings
☒	☐	For hierarchical and complex designs, identification of the appropriate level for tests and full reporting of outcomes
☒	☐	Estimates of effect sizes (e.g. Cohen's *d*, Pearson's *r*), indicating how they were calculated

Our web collection on statistics for biologists contains articles on many of the points above.

Software and code

Policy information about availability of computer code

Data collection	SSRF beamlines BL17U1, BL18U1 and BL19U1
Data analysis	XDS Program Package 2: Diffraction data reduction<br>HKL2000 v708: X-ray data processing<br>WinCoot 0.8.6.1: Protein model building<br>PHASER in PHENIX 1.19.2-4158: molecular replacement, structure determination and refinement<br>Biacore T200 Evaluation Software 3.1, Microsoft Excel 2016 and GraphPad Prism 8.0: Data analysis<br>PyMOL 2.5.2: Molecular graphics and analysis

For manuscripts utilizing custom algorithms or software that are central to the research but not yet described in published literature, software must be made available to editors and reviewers. We strongly encourage code deposition in a community repository (e.g. GitHub). See the Nature Portfolio guidelines for submitting code & software for further information.

Data

Policy information about availability of data

All manuscripts must include a data availability statement. This statement should provide the following information, where applicable:

- Accession codes, unique identifiers, or web links for publicly available datasets
- A description of any restrictions on data availability
- For clinical datasets or third party data, please ensure that the statement adheres to our policy

The data that support the findings of this study are available from the corresponding author upon reasonable request. The coordinates and structure factors have been deposited in the Protein Data Bank under accession codes 8GP0 [https://doi.org/10.2210/pdb8GP0/pdb], 8GOL [https://doi.org/10.2210/pdb8GOL/pdb], 7Y0L [https://doi.org/10.2210/pdb7Y0L/pdb], 8IVN [https://doi.org/10.2210/pdb8IVN/pdb], 8IW3 [https://doi.org/10.2210/pdb8IW3/pdb], 8IW6 [https://doi.org/10.2210/pdb8IW6/pdb], 8IVS [https://doi.org/10.2210/pdb8IVS/pdb], 8IVT [https://doi.org/10.2210/pdb8IVT/pdb], 8J7J [https://doi.org/10.2210/pdb8J7J/pdb], 8J7G [https://doi.org/10.2210/pdb8J7G/pdb], 8GOY [https://doi.org/10.2210/pdb8GOY/pdb], 7YD2 [https://doi.org/10.2210/pdb7YD2/pdb], 8IVM [https://doi.org/10.2210/pdb8IVM/pdb], 8IVE [https://doi.org/10.2210/pdb8IVE/pdb] and 8J7K [https://doi.org/10.2210/pdb8J7K/pdb]. Source data are provided with this paper.

Human research participants

Policy information about studies involving human research participants and Sex and Gender in Research.

Reporting on sex and gender	n/a
Population characteristics	n/a
Recruitment	n/a
Ethics oversight	n/a

Note that full information on the approval of the study protocol must also be provided in the manuscript.

Field-specific reporting

Please select the one below that is the best fit for your research. If you are not sure, read the appropriate sections before making your selection.

[x] Life sciences [ ] Behavioural & social sciences [ ] Ecological, evolutionary & environmental sciences

For a reference copy of the document with all sections, see nature.com/documents/nr-reporting-summary-flat.pdf

Life sciences study design

All studies must disclose on these points even when the disclosure is negative.

Sample size	No statistica methods were used to predetermine sample size, the size of crystals sample is determined by protein stability and crystallization conditions.Crystallographic data were collected for 2-3 crystals diffracting X-rays to the highest resolution and were analyzed to ensure the highest quality of the structural data. The individual mutants were assayed for SulE de-esterification activity in three independent activity measurements. Sample sizes (replicates) were chosen based on our previous experience with published work in enzymology (J. Agric. Food Chem. 67, 836-843 (2019)).
Data exclusions	No data were excluded from the analysis.
Replication	All enzymatic experiments were repeated in 3 times as described in figure legends. Crystalization trials have been successfully repeated at least 5 times with reproducibly diffracting crystals.
Randomization	Randomizaticn was not relevant to this study since the study does not involve different experimental groups and was not an animal study or clinical trial.
Blinding	Blinding was not used in this study because data were derived from instrument-based measurement and software-based analysis with minimal risk of bias.

Reporting for specific materials, systems and methods

We require information from authors about some types of materials, experimental systems and methods used in many studies. Here, indicate whether each material, system or method listed is relevant to your study. If you are not sure if a list item applies to your research, read the appropriate section before selecting a response.

Materials & experimental systems

n/a	Involved in the study	
☒	☐	Antibodies
☒	☐	Eukaryotic cell lines
☒	☐	Palaeontology and archaeology
☒	☐	Animals and other organisms
☒	☐	Clinical data
☒	☐	Dual use research of concern

Methods

n/a	Involved in the study	
☒	☐	ChIP-seq
☒	☐	Flow cytometry
☒	☐	MRI-based neuroimaging
